# Correction: A glial cell-derived pathway directs regenerating optic nerve axons toward the optic chiasm

**DOI:** 10.1242/dev.205860

**Published:** 2026-06-25

**Authors:** Beth M. Harvey, Melissa Baxter, Alexis M. Garcia, Michael Granato

There was an error in *Development* (2026) **153**, dev205048 (doi:10.1242/dev.205048).

In the ‘Fish lines and maintenance’ section of the Materials and Methods, c*ol7a1^sa3473 ^*was listed instead of *col7a1^sa6935^*. The original and corrected text are shown below.

**Corrected:**

The following transgenic lines and mutants were used: *lh3^TV2O5^* (Schneider and Granato, 2006), *col18a1* (Isaacman-Beck et al., 2015), *col4a5^s510^* (Xiao and Baier, 2007), *col7a1^sa6935^*, *Tg(hsp:lh30-myc)* (Isaacman-Beck et al., 2015), *Tg(sox10:lh3-mkate)* (Isaacman-Beck et al., 2015), *Tg(isl2b:GFP)* (Pittman et al., 2008), *Tg(olig2:dsred)* (Kucenas et al., 2008), *Tg(mpeg1:Gal4FF)^gl25^* (Ellett et al., 2011) and *Tg(UAS:nfsB-mCherry)^c264^* (Davison et al., 2007).

**Original:**

The following transgenic lines and mutants were used: *lh3^TV2O5^* (Schneider and Granato, 2006), *col18a1* (Isaacman-Beck et al., 2015), *col4a5^s510^* (Xiao and Baier, 2007), c*ol7a1^sa3473^*, *Tg(hsp:lh30-myc)* (Isaacman-Beck et al., 2015), *Tg(sox10:lh3-mkate)* (Isaacman-Beck et al., 2015), *Tg(isl2b:GFP)* (Pittman et al., 2008), *Tg(olig2:dsred)* (Kucenas et al., 2008), *Tg(mpeg1:Gal4FF)^gl25^* (Ellett et al., 2011) and *Tg(UAS:nfsB-mCherry)^c264^* (Davison et al., 2007).

Both the online full-text and PDF versions of the article have been updated.

This correction does not affect the results in the article or the conclusions of this study. The authors apologise for this error and any inconvenience it may have caused.

